# *O*-GlcNAcylation Links Nutrition to the Epigenetic Downregulation of *UNC5A* during Colon Carcinogenesis

**DOI:** 10.3390/cancers12113168

**Published:** 2020-10-28

**Authors:** Amélie Decourcelle, Ninon Very, Madjid Djouina, Ingrid Loison, Julien Thévenet, Mathilde Body-Malapel, Eric Lelièvre, Olivier Coqueret, Dominique Leprince, Ikram El Yazidi-Belkoura, Vanessa Dehennaut

**Affiliations:** 1 Université de Lille, CNRS, Inserm, CHU Lille, UMR9020-U1277—CANTHER—Cancer Heterogeneity, Plasticity and Resistance to Therapies, F-59000 Lille, France; amelie.decourcelle@ibl.cnrs.fr (A.D.); ingrid.loison@ibl.cnrs.fr (I.L.); dominique.leprince@ibl.cnrs.fr (D.L.); 2 Université de Lille, CNRS, UMR 8576—UGSF—Unité de Glycobiologie Structurale et Fonctionnelle, F-59000 Lille, France; ninon.very.etu@univ-lille.fr (N.V.); ikram.el-yazidi@univ-lille.fr (I.E.Y.-B.); 3 Université de Lille, Inserm, CHU Lille, U1286—INFINITE—Institute for translational research in inflammation, F-59000 Lille, France; madjid.djouina@univ-lille.fr (M.D.); mathilde.body@univ-lille.fr (M.B.-M.); 4 Université de Lille, Inserm, CHU Lille, UMR 1190 Translational Research for Diabetes, European Genomic Institute for Diabetes, F-59000 Lille, France; julien.thevenet@univ-lille.fr; 5 Paul Papin ICO Cancer Center, CRCINA, INSERM, Université de Nantes, Université d’Angers, 49055 Angers, France; eric.lelievre@univ-angers.fr (E.L.); olivier.coqueret@univ-angers.fr (O.C.)

**Keywords:** UNC5A, *O*-GlcNAcylation, OGT, EZH2, nutrition, epigenetics, colon cancer

## Abstract

**Simple Summary:**

Nutritional disorders represent major risk factors for colorectal cancer according to mechanisms of action that are still insufficiently known. The aim of our study was to investigate the putative involvement of nutrition in the epigenetic downregulation of the tumor suppressor genes of the *UNC5* (Uncoordinated 5) family during colonic carcinogenesis and to understand its molecular relays. Herein, we provided evidence that the consumption of a High Carbohydrate Diet worsens colon carcinogenesis in mice and is correlated with the downregulation of several members of the *UNC5* family whose *UNC5A* (Uncoordinated 5A). Mechanistically, we identified the nutritional sensor *O*-GlcNAcylation as one of the molecular relays that regulate the recruitment of the PRC2 complex onto the *UNC5A* promoter to repress its transcription.

**Abstract:**

While it is now accepted that nutrition can influence the epigenetic modifications occurring in colorectal cancer (CRC), the underlying mechanisms are not fully understood. Among the tumor suppressor genes frequently epigenetically downregulated in CRC, the four related genes of the *UNC5* family: *UNC5A*, *UNC5B*, *UNC5C* and *UNC5D* encode dependence receptors that regulate the apoptosis/survival balance. Herein, in a mouse model of CRC, we found that the expression of *UNC5A*, *UNC5B* and *UNC5C* was diminished in tumors but only in mice subjected to a High Carbohydrate Diet (HCD) thus linking nutrition to their repression in CRC. *O*-GlcNAcylation is a nutritional sensor which has enhanced levels in CRC and regulates many cellular processes amongst epigenetics. We then investigated the putative involvement of *O*-GlcNAcylation in the epigenetic downregulation of the *UNC5* family members. By a combination of pharmacological inhibition and RNA interference approaches coupled to RT-qPCR (Reverse Transcription-quantitative Polymerase Chain Reaction) analyses, promoter luciferase assay and CUT&RUN (Cleavage Under Target & Release Using Nuclease) experiments, we demonstrated that the *O*-GlcNAcylated form of the histone methyl transferase EZH2 (Enhancer of Zeste Homolog 2) represses the transcription of *UNC5A* in human colon cancer cells. Collectively, our data support the hypothesis that *O*-GlcNAcylation could represent one link between nutrition and epigenetic downregulation of key tumor suppressor genes governing colon carcinogenesis including *UNC5A*.

## 1. Introduction

The emergence and progression of cancer depend on a complex interplay between the genome and the epigenome, which together interact with several environmental factors. Particularly, several studies have highlighted the key role of diet and nutritional compounds in the epigenetic regulation of gene expression [1,2,3]. They hence support the hypothesis of a close relationship between nutritional disorders (obesity, metabolic syndrome, type 2 diabetes, etc.), well-known risk factors for many cancers including colorectal cancer (CRC), and epigenetic reprogramming linked to carcinogenesis. 

The *UNC5* gene family consists of four related genes including *UNC5A*, *UNC5B*, *UNC5C* and *UNC5D* that encode type-I transmembrane receptors of Netrin-1. UNC5 and Netrin-1 play essential role in axon guidance during neuronal development and differentiation [4]. In addition, in neuronal and non-neuronal cells, UNC5 receptors share the capability to act as “dependence receptors”: they transduce a “positive” cell proliferation and survival signal when bound to Netrin-1 but induce caspase-dependent apoptosis in absence of their ligand. In recent years, the UNC5 receptors have been defined as key players of colorectal carcinogenesis by regulating the survival/apoptosis balance and are considered as conditional tumor suppressor genes [5]. In fact, expression of *UNC5A*, *UNC5B* and *UNC5C* is frequently downregulated in colorectal cancer (CRC) and their silencing has been associated in part with loss of heterozygoty (LOH) within *UNC5* loci and with epigenetic alterations that are not fully understood [6,7,8,9,10]. Notably, the putative influence of nutrition on the repression of the *UNC5* family members during colon carcinogenesis has not yet been investigated.

Among the molecular elements that could connect nutrition to epigenetic reprogramming in CRC, the nutritional sensor *O*-linked-β-N-acetylglucosaminylation (*O*-GlcNAcylation) has emerged during the last decade as a key regulator of chromatin remodeling and thereby of the epigenetic regulation of gene expression [11,12,13,14]. *O*-GlcNAcylation is a reversible post-translational modification of nucleocytoplasmic and mitochondrial proteins that consists in the covalent linkage of a unique sugar N-acetylglucosamine (GlcNAc) to serines and threonines of target proteins [15]. *O*-GlcNAcylation levels are regulated by a unique couple of enzymes: OGT (*O*-GlcNAc Transferase) that catalyzes the transfer of GlcNAc from UDP-GlcNAc onto the protein and OGA (*O*-GlcNAcase) that hydrolyzes the residue. *O*-GlcNAcylation levels are closely dependent upon the concentration of UDP-GlcNAc synthetized through the Hexosamine Biosynthesis Pathway (HBP) at the crossroad of glucose, amino acid, fatty acid and nucleotide metabolisms. UDP-GlcNAc and *O*-GlcNAcylation are thus considered as sensors of the nutritional state which can relay the effects of an excessive food supply, unbalanced diet, obesity and other metabolic problems that represent high risk factors of CRC [16,17,18]. In this sense, through Western Blot analyses, we previously showed that colons from mice fed a High Carbohydrate Diet (HCD) exhibited higher amounts of *O*-GlcNAcylation relative to mice fed a Normal Diet (ND) [19]. Moreover, we and others observed increased contents of *O*-GlcNAcylation and OGT in human colon cancer samples in comparison with normal tissues [19,20,21,22,23]. Contrary, decreasing *O*-GlcNAcylation levels by silencing OGT reduces proliferation, adhesion, migration and anchorage-independent cell growth of colon cancer cell lines [20,24]. Aberrant OGT and *O*-GlcNAcylation levels are thus defined as new CRC hallmarks [25]. 

*O*-GlcNAcylation is involved in the regulation of many cellular processes, including the epigenetic regulation of gene expression. Indeed, *O*-GlcNAcylation is part of the histone code and OGT interacts with and regulates the DNA demethylases of the Ten-Eleven-Translocation (TET) family and several histone modifying proteins [11,12,13]. Among this last class of proteins, the members of the Polycomb Repressive Complex 2 (PRC2) repress the transcription of numerous target genes through the deposit of the repressive chromatin mark H3K27Me3 consisting of trimethylation of lysine 27 on histone H3. The core of PRC2 is composed of three Polycomb group (PcG) proteins: Enhancer of Zeste Homolog 2 (EZH2), Embryonic Ectoderm Development (EED) and Suppressor of Zeste 12 (SUZ12). The methyl transferase EZH2 is the catalytic subunit of the PRC2 complex and SUZ12 and EED are indispensable for EZH2 enzymatic activity. Several studies have reported abnormally elevated expression of EZH2, EED and SUZ12 in CRC in correlation with advanced stages of the disease and poor prognosis [26,27,28]. Regulation of EZH2 functions by its *O*-GlcNAcylation has been evidenced in several studies conducted in different cell lines including breast and colon cancer cells [29,30,31,32,33]. In the colon cancer cell line HT29, *O*-GlcNAcylated proteins and H3K27 trimethylation were found together at the promoter region of 61 genes [34]. In breast cancer MCF7 cells, a co-regulation by OGT and EZH2 was also evidenced for 16 tumor suppressor genes including *UNC5A* [29]. However, the involvement of this OGT-EZH2 axis in the regulation of the expression of *UNC5A* as well as the other members of the *UNC5A* family in colon cancer cells has not been studied. 

Therefore, in this study, we investigated whether nutrition could influence the expression of the *UNC5* family members during colon carcinogenesis and whether it could be related to the OGT-EZH2 axis.

## 2. Results

### 2.1. Subjecting Mice to a High Carbohydrate Diet (HCD) Worsens Colon Carcinogenesis

**Figure 1:**
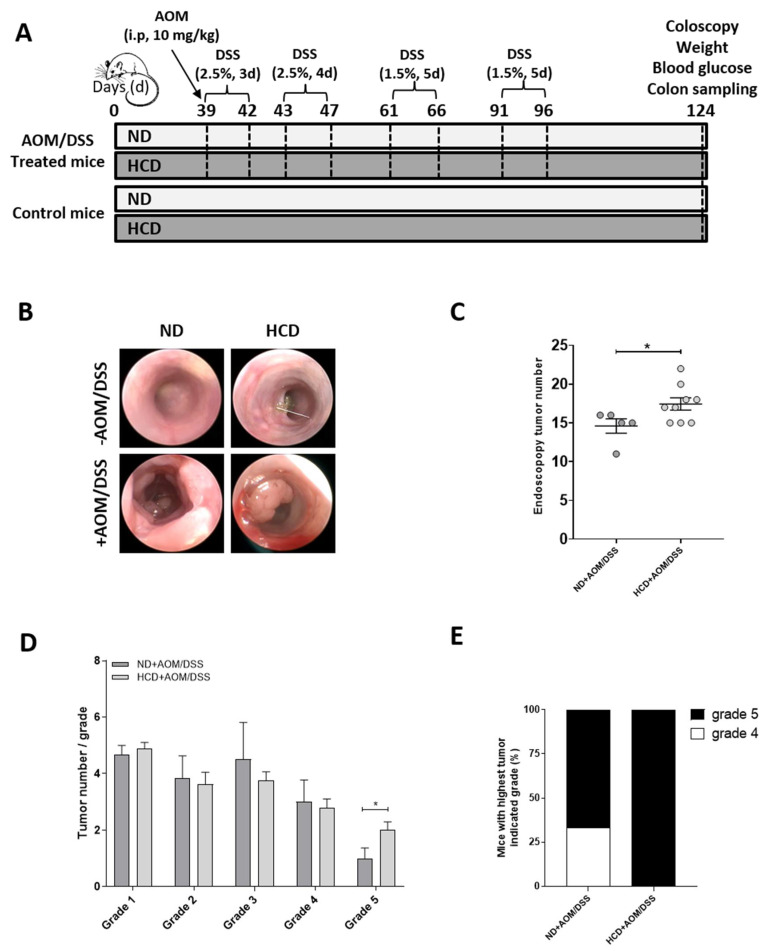

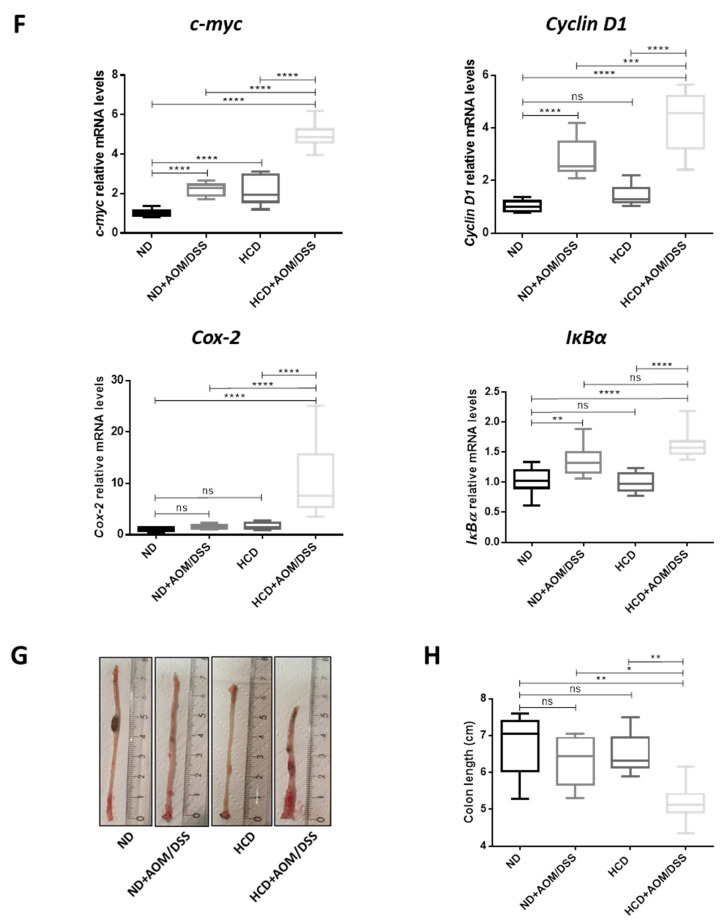
Subjecting mice to a High Carbohydrate Diet (HCD) worsens colon carcinogenesis. (**A**) Experimental protocol. Half of the C57BL/6JRj mice were subjected to a Normal Diet (ND) and the other half to a High Carbohydrate Diet (HCD). Thirty-nine days after the beginning of the two diets, half of the experimental animals received a single intraperitoneal injection of azoxymethane (AOM, 10 mg/kg) and they began to receive 2.5% dextran sulfate sodium (DSS) in drinking water for 3 consecutive days followed by drinking water for 1 day. Mice were then submitted to three additional cycles of DSS (4 days with 2.5% DSS for the first one and 5 days with 1.5% DSS for the two others) with a resting period of 14 days between each cycle. Animals were sacrificed 85 days after AOM injection (day 124). At this time, each group contained the following number of animals: ND (n = 5), ND + AOM/DSS (n = 6), HCD (n = 7) and HCD + AOM/DSS (n = 9). At day 124, tumor burden was monitored via endoscopy (**B**) and the total tumors numbers (**C**), the number of tumors per grade (**D**) and the percentage of mice ranked by the most severe grade (**E**) were then determined. * *p* < 0.05: student’s *t*-test. (**F**) Relative expression of c-myc, Cyclin D1, Cox-2 and IkBα were assessed by RT-qPCR. Values are normalized to RPLP0. ns: non-significant, ** *p* < 0.01, *** *p* < 0.001, **** *p* < 0.0001: Bonferroni’s multiple comparisons test. (**G**–**H**) Colon length was determined just after surgery. ns: non-significant, * *p* < 0.05, ** *p* < 0.01: Bonferroni’s multiple comparisons test.

To test whether nutrition could be involved in the epigenetic downregulation of *UNC5* receptors during colon carcinogenesis, we subjected C57BL/6JRj mice either to a Normal Diet (ND) or to a High Carbohydrate Diet (HCD). Thirty-nine days after the beginning of the different diets, we induced CRC in these mice using the well-characterized azoxymethane (AOM)/dextran sulfate sodium (DSS) method [35] (Figure 1A). At the end of experiment, mice treated with AOM/DSS and fed HCD had a statistically significant higher blood glucose level compared to mice treated with AOM/DSS and fed ND (Supplementary Figure S1A). Moreover, weight loss was observed in mice treated with AOM/DSS and fed HCD (Supplementary Figure S1B) probably due to the severity of the disease in this group of animals. Indeed, we monitored tumor burden via endoscopy (Figure 1B) and observed that mice fed HCD had a higher number of tumors than the control group (Figure 1C) with a higher number of grade 5 tumors (Figure 1D) observed in 100% of mice (Figure 1E). We also studied the expression of *c-myc* and *cyclin D1*, two well-known target genes of the Wnt/β-catenin pathway, whose activity is commonly upregulated in CRC. As expected, both *c-myc* and *Cyclin D1* transcripts were increased in tumors in mice fed ND compared to the control group (Figure 1F, compare ND vs. ND + AOM/DSS). Interestingly, the High Carbohydrate Diet caused an even greater increase in *c-myc* and *Cyclin D1* expression (Figure 1F, compare ND + AOM/DSS vs. HCD + AOM/DSS). Moreover, in mice treated with AOM/DSS in conjunction with the HCD, we also observed a clear decrease in colon length compared to mice fed ND (Figure 1G,H) and an increase in *Cox-2* and *IκBα* mRNA levels (that indicates activation of the NF-κB pathway) (Figure 1F) thus reflecting a higher level of inflammation in these mice. Taken together, these results demonstrate, as we might expect, that the High Carbohydrate Diet worsens inflammation-driven colon carcinogenesis in mice. 

### 2.2. UNC5A, UNC5B and UNC5C Expression Is Downregulated in Colon Tumors Only in Mice Subjecting to the High Carbohydrate Diet

**Figure 2:**
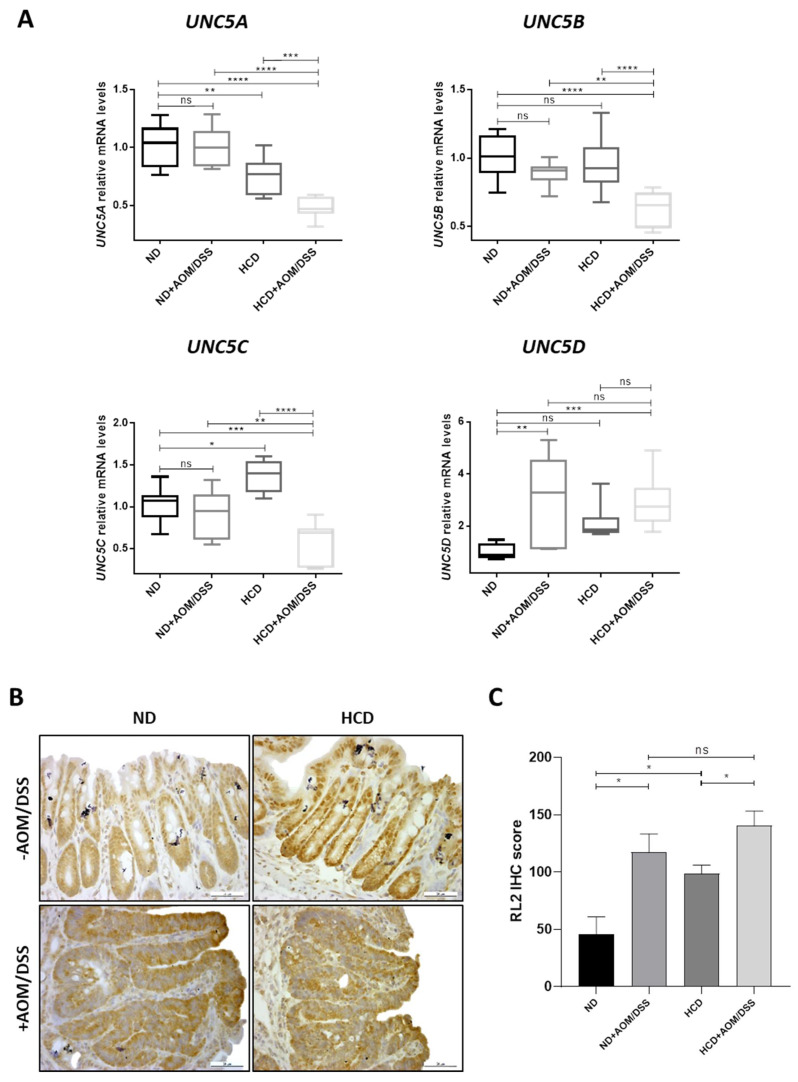
*UNC5A*, *UNC5B* and *UNC5C* expression is downregulated in colon tumors only in mice subjected to a High Carbohydrate Diet in association with an increase in O-GlcNAcylation levels. (**A**) Relative expression of UNC5A, UNC5B, UNC5C and UNC5D were assessed by RT-qPCR in the colon of mice fed a normal diet (ND) or a High Carbohydrate Diet (HCD) in combination or not with AOM/DSS. Values are normalized to RPLP0. ns: non-significant; * *p* < 0.05, ** *p* < 0.01, *** *p* < 0.001, **** *p* < 0.0001: Bonferroni’s multiple comparisons test. (**B**) Tissue samples processed for immunohistochemistry staining of RL2. Pictures shown are representative of two sections per mouse. (**C**) IHC score performed in (**B**) was scored as described in the methods section and plotted as mean ± sem. * *p* < 0.05: unpaired *t*-test.

We then examined the colonic expression of UNC5A, UNC5B, UNC5C and UNC5D by RT-qPCR analysis (Figure 2A). Surprisingly and in disagreement with their tumor suppressor gene status, we did not observe any difference in the expression of UNC5A, UNC5B, and UNC5C between control group (ND) and AOM/DSS group under Normal Diet (ND + AOM/DSS) and UNC5D was found to increase (Figure 2A). These results suggest that downregulation of these family of genes is not necessary for CRC emergence. In contrast, our results showed a marked decrease in the level of UNC5A, UNC5B and UNC5C transcripts in the colon of AOM/DSS treated mice fed HCD (Figure 2A, compare ND or ND + AOM/DSS vs. HCD + AOM/DSS) strongly suggesting that nutrition is involved in the epigenetic downregulation of these genes that might occur during the progression of CRC. 

### 2.3. O-GlcNAcylation Levels Are Enhanced in Colon from Mice Fed HCD and in Response to AOM/DSS Treatment

To test whether *O*-GlcNAcylation could be one of the molecular relays between this nutrition-dependent downregulation of UNC5A, UNC5B and UNC5C in AOM/DSS treated mice fed HCD, we performed immunohistochemistry experiments (Figure 2B,C). A 2.5 fold increase in IHC (Immunohistochemistry) score in tumors compared to normal tissues were observed in mice fed ND thus demonstrating that enhanced *O*-GlcNAcylation levels seem to be also a hallmark of CRC in mice (Figure 2B,C, compare ND vs. ND + AOM/DSS). In non-tumorigenic mice colons, the results showed that the HCD caused a 2 fold increase in colonic *O*-GlcNAcylation levels compared to the normal diet (Figure 2B,C, compare ND vs. HCD) thus confirming our previous work [19]. In addition, *O*-GlcNAcylation levels are further increased when the HCD is combined to the AOM/DSS treatment (Figure 2B,C, compare HCD vs. HCD + AOM/DSS). Nevertheless, the *O*-GlcNAcylation levels are not statistically higher in tumorigenic mice fed HCD compared to ND. This last observation argues against a direct link between *O*-GlcNAcylation levels and the downregulation of the members of the UNC5 family observed in tumorigenic mice fed HCD. However, and in an inverse correlation with the *O*-GlcNAcylation levels, our results show that subjecting mice to the High Carbohydrate Diet is sufficient to decrease the expression of UNC5A but not of the other members of the *UNC5* family in non-tumorigenic mice colons (Figure 2A, compare ND vs. HCD) and that this decrease in UNC5A transcripts is even greater in mice treated with AOM/DSS and subjected to the HCD (Figure 2A, compare HCD vs. HCD + AOM/DSS). Therefore, we hypothesized that *O*-GlcNAcylation could be one of the molecular relays between High Carbohydrate Diet and downregulation of the colonic expression of UNC5A exclusively.

### 2.4. O-GlcNAcylation Is Involved in the Regulation of UNC5A Gene Expression in Human Colon Cancer HCT116 Cells

**Figure 3:**
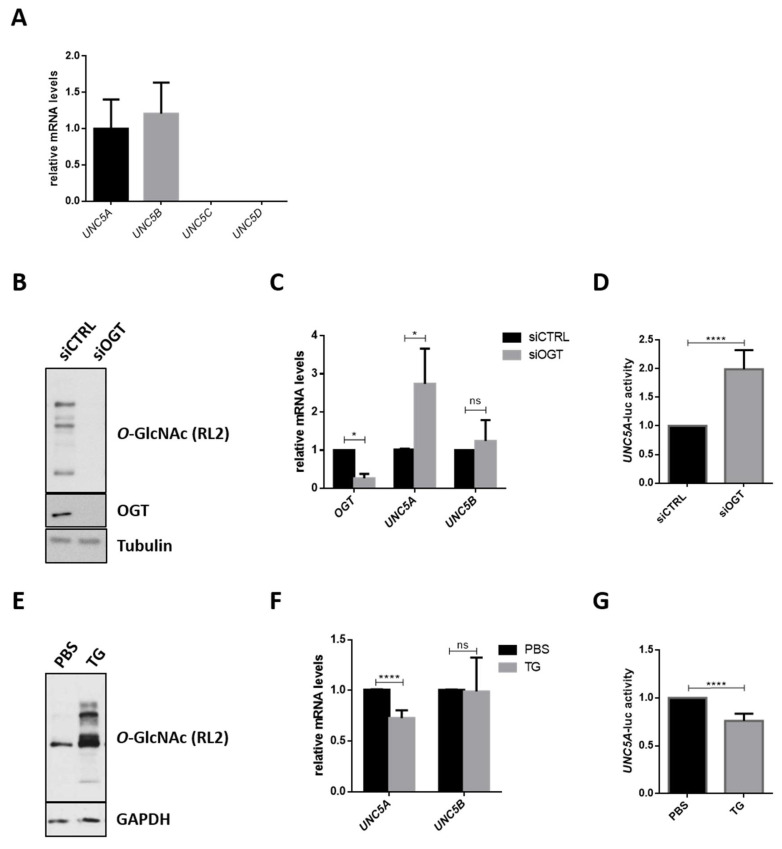
*O*-GlcNAcylation is involved in the regulation of the transcription of *UNC5A* in human colon cancer HCT116 cells. (**A**) Expression of the four members of the *UNC5* gene family (*UNC5A*, *UNC5B*, *UNC5C* and *UNC5D*) was analyzed by RT-qPCR in HCT116 cells. Data shown are the average ± SD of three independent experiments. (**B**–**D**) HCT116 cells were transfected with non-target control siRNA (siCTRL) or with OGT siRNA (siOGT) for 72 h. (**B**) To ensure siRNA efficiency, a fraction of the cell lysates was analyzed by Western blot (WB) with anti-*O*-GlcNAc (RL2) and anti-OGT antibodies. Tubulin was used as a loading control. Data shown are representative of three independent experiments. (**C**) Cells were harvested for total RNA extraction. The mRNA expression levels of OGT, UNC5A and UNC5B were assessed by RT-qPCR. Values were normalized to *RPLP0*. Data shown are the average ± SD of three independent experiments. ns: non-significant, * *p* < 0.05: unpaired *t*-test. (**D**) Then, 24 h after siRNA transfection, cells were transfected with 200 ng of an *UNC5A* promoter activity reporter plasmid and 25 ng of β-galactosidase reporter. Thus, 48 h later, *UNC5A*-luciferase activity was determined as described in the experimental procedures section. Data shown are the average ± SD of three independent experiments. **** *p* < 0.0001: unpaired *t*-test. (**E**–**G**) HCT116 cells were treated with the OGA inhibitor Thiamet G (TG) (1 µM final in PBS (Phosphate Buffered Saline)) for 24 h. (**E**) Treatment efficiency was ensured by WB analyses of *O*-GlcNAcylation (RL2) levels. GAPDH (Glyceraldehyde-3-phosphate dehydrogenase) was used as a loading control. Data shown are representative of three independent experiments. (**F**) mRNA expression level of UNC5A and UNC5B were assessed by RT-qPCR. Values were normalized to *RPLP0*. Data shown are the average ± SD of three independent experiments. **** *p* < 0.0001: unpaired *t*-test. (**G**) Cells were transfected with 200 ng of an *UNC5A* promoter activity reporter plasmid and 25 ng of β-galactosidase reporter. Following this, 24 h later, cells were treated with TG (1 µM final in PBS) for 24 h. *UNC5A*-luciferase activity was determined as described in the experimental procedures section. Data shown are the average ± SD of three independent experiments. **** *p* < 0.0001: unpaired *t*-test.

To evaluate the role of *O*-GlcNAcylation in the epigenetic downregulation of the *UNC5* family, we first examined the expression of UNC5A, UNC5B, UNC5C and UNC5D transcripts in the human colon cancer cell line HCT116 (Figure 3A). We detected significant levels of only UNC5A and UNC5B transcripts. We then investigated the effect of the knock-down of OGT by siRNA on the levels of the *UNC5* family members in this cell line. siOGT efficiency was checked at protein (Figure 3B) and mRNA level (Figure 3C) and we found that it induced a significant increase in UNC5A mRNA levels but had no effect on UNC5B (Figure 3C). Moreover, UNC5C and UNC5D transcripts remained undetectable upon OGT silencing (Decourcelle, A.; Dehennaut, V.; Université de Lille, CNRS, Inserm, CHU Lille, UMR9020-U1277—CANTHER—Cancer Heterogeneity, Plasticity and Resistance to Therapies, F-59000 Lille, France. Personal observation, 2020.). This first observation supports our hypothesis that *O*-GlcNAcylation is only involved in the regulation of the expression of UNC5A but not of the other members of the family. mRNA amounts can be regulated either at the transcriptional level or at the post-transcriptional level (mRNA stability). In order to investigate whether siOGT-induced increase of *UNC5A* expression was due to transcriptional regulation, we used a previously described *UNC5A* promoter activity reporter plasmid [36,37] in luciferase assay. As shown in Figure 3D, siOGT caused a 2-fold induction of the *UNC5A* promoter activity correlated with a decrease in *O*-GlcNAcylation levels (Figure 3B). Inversely, treating HCT116 cells with the potent OGA inhibitor Thiamet G (TG) induced an *O*-GlcNAcylation increase (Figure 3E) correlated with a drop of 25% in UNC5A mRNA levels (Figure 3F) and promoter activity (Figure 3G). In contrast, Thiamet G treatment has no effect on UNC5B mRNA levels (Figure 3F). Taken together, these data demonstrate that *O*-GlcNAcylation is involved in the regulation of the transcription of colonic *UNC5A*.

### 2.5. O-GlcNAcylation Regulates the PRC2-Mediated Repression of UNC5A in Human Colon Cancer Cells

**Figure 4:**
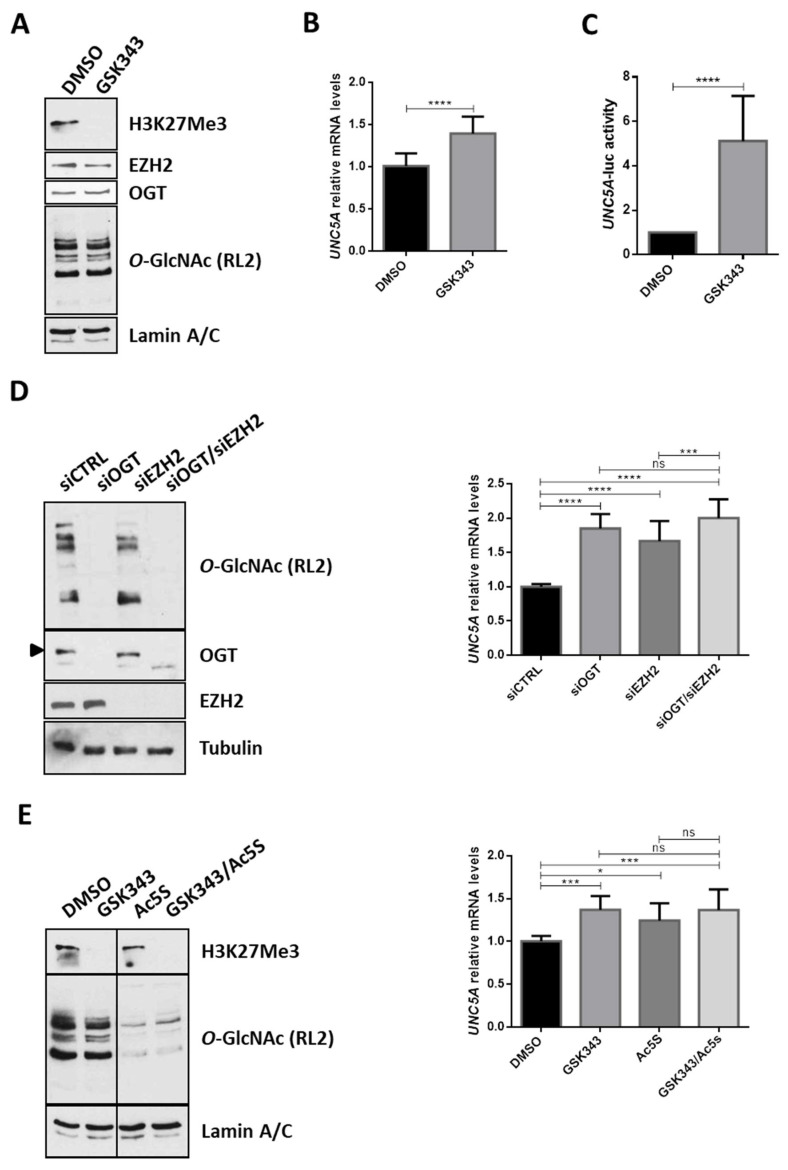
EZH2 and OGT act together to repress the transcription of *UNC5A* in colon cancer cells. (**A**,**B**) HCT116 cells were treated with the EZH2 inhibitor GSK343 (5 µM final in DMSO (Dimethyl sulfoxide)) for 48 h. (**A**) Treatment efficiency was ensured by WB analyses of H3K27Me3 levels from chromatin-bound proteins extracts. EZH2, OGT and *O*-GlcNAcylation (RL2) levels were also assessed. Lamin A/C were used as a loading control. Data shown are representative of three independent experiments. (**B**) mRNA expression level of UNC5A was assessed by RT-qPCR. Values were normalized to RPLP0. Data shown are the average ± SD of three independent experiments. **** *p* < 0.0001: unpaired *t*-test. (**C**) Cells were transfected with 200 ng of an *UNC5A* promoter activity reporter plasmid and 25 ng of β-galactosidase reporter. Thus, 6 h later, cells were treated with GSK343 (5 µM final in DMSO) for 48 h. *UNC5A*-luciferase activity was determined as described in the experimental procedures section. Data shown are the average ± SD of three independent experiments. **** *p* < 0.0001: unpaired *t*-test. (**D**) HCTT116 cells were transfected either with non-target siRNA control (siCTRL), OGT siRNA (siOGT), EZH2 siRNA (siEZH2) or with a combination of siOGT and siEZH2 for 72 h. Cells were harvested either for total proteins or RNA extraction. Left panel: WB analyses were performed using the indicated antibodies. Data shown are representative of five independent experiments. Right panel: mRNA expression level of *UNC5A* was assessed by RT-qPCR. Values were normalized to *RPLP0*. Data shown are the average ± SD of five independent experiments. ns: non-significant, *** *p* < 0.001, **** *p* < 0.0001: Bonferroni’s multiple comparisons test. (**E**) HCTT116 cells were treated with the EZH2 inhibitor GSK343 (5 µM final in DMSO) or the OGT inhibitor Ac5S-GlcNAc (50 µM final in DMSO) alone or in combination. Cells were harvested either for total protein or RNA extraction. Left panel: WB analyses of Lamin A/C, H3K27Me3 and *O*-GlcNAcylation levels from chromatin-bound proteins extracts. Data shown are representative of four independent experiments. Right panel: amount of *UNC5A* transcripts was assessed by RT-qPCR. Values were normalized to *RPLP0*. Data shown are the average ± SD of four independent experiments. ns: non-significant, * *p* < 0.05, *** *p* < 0.001: Bonferroni’s multiple comparisons test.

**Figure 5:**
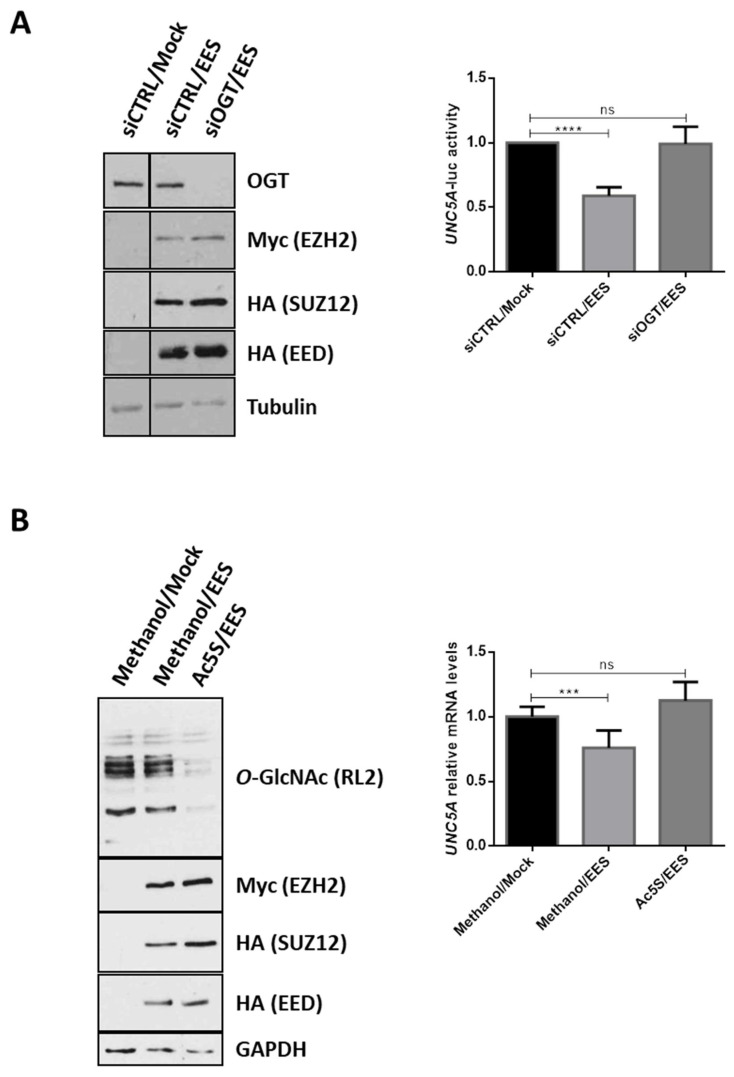
*O*-GlcNAcylation mediates the PRC2-mediated repression of *UNC5A*. (**A**) HCT116 cells were co-transfected with the core PRC2 complex (EES: Myc-EZH2, HA-EED, HA-SUZ12), 200 ng of plasmid encoding the luciferase reporter gene under the control of the *UNC5A* promoter and 25 ng of β-galactosidase in combination or not with siOGT. Left panel: transfection efficiency and OGT inhibition were checked by WB analyses with the indicated antibodies. Data shown are representative of three independent experiments. Right panel: luciferase activity was determined 48 h later and was normalized to β-galactosidase activity. Data shown are the average ± SD of three independent experiments. ns: non-significant, **** *p* < 0.0001: Bonferroni’s multiple comparison tests. (**B**) Cells were co-transfected with plasmids encoding the core PRC2 (EES: Myc-EZH2, HA-EED, HA-SUZ12), or with empty vectors (Mock). Thus, 24 h after transfection, cells were treated with the potent OGT inhibitor Ac5s-GlcNAc (50 µM final in methanol) for 24 h. Left panel: transfection efficiency and OGT inhibition were checked by WB analyses with the indicated antibodies. Data shown are representative of three independent experiments. Right panel: mRNA expression level of UNC5A was assessed by quantitative RT-PCR. Values were normalized to RPLP0. Data shown are the average ± SD of three independent experiments. ns: non-significant, *** *p* < 0.001: Bonferroni’s multiple comparison tests.

As mentioned in the introduction section, several members of the Polycomb Repressive Complex 2 (PRC2), including the histone methyl transferase EZH2, are overexpressed in CRC [26,27,28]. Moreover, several studies have demonstrated that OGT and *O*-GlcNAcylation regulate EZH2 functions [29,30,31,32,33]. Next, we wondered whether the PRC2 complex was also involved in the regulation of *UNC5A* transcription in colon cancer cells. To answer this question, we first inhibited the methyl transferase activity of EZH2 in HCT116 cells with GSK343, a selective SAM (S-adenosyl methionine)-competitive EZH2 inhibitor (Figure 4A) and analyzed the levels of UNC5A mRNA (Figure 4B) and promoter activity (Figure 4C). GSK343 clearly diminished the level of H3K27 trimethylation in cells (Figure 4A) and increased *UNC5A* expression (Figure 4B) and promoter activity (Figure 4C). These first results thus suggest that EZH2 is also involved in the regulation of *UNC5A* expression in HCT116 cells. To confirm this result, we performed siRNA knockdown of EZH2 alone or in combination with siOGT in HCT116 cells and analyzed the levels of UNC5A mRNA (Figure 4D). We first observed that siEZH2 induced a derepression of *UNC5A* expression in line with the results obtained with GSK343 inhibitor. Moreover, siEZH2 increased UNC5A transcripts at a level similar to siOGT and the simultaneous knockdown of OGT and EZH2 had no additive effect compared to siEZH2 or siOGT alone. It is also to note that the abolition of the expression of EZH2 or the inhibition of its catalytic activity had no effect on OGT expression nor on *O*-GlcNAcylation levels (Figure 4A,D,E) arguing against an indirect modulation of *UNC5A* expression through the modulation of OGT functions in these conditions. This last result thus suggests that OGT and EZH2 act together to repress *UNC5A* transcription. We confirmed this result by inhibiting OGT and/or EZH2: respectively with Ac5S-GlcNAc (Ac5S) and GSK343 specific inhibitors (Figure 4E) and validated them also in LS174T human colorectal cancer cell line (Supplementary Figure S2). We then overexpressed the catalytic core of PRC2 complex (composed of EZH2, SUZ12 and EED) in HCT116 cells by co-transfecting cells with plasmids encoding Myc-EZH2, HA-SUZ12 and HA-EED (Figure 5A, EES). In PRC2-transfected cells, we observed a 40% reduction of the *UNC5A* promoter activity compared to mock-transfected ones (Figure 5A, right panel) thus confirming that *UNC5A* is a target of the PRC2 complex. However, this repression was no longer observed in cells co-transfected with siOGT suggesting that *O*-GlcNAcylation is required for the PRC2-mediated repression of *UNC5A*. In a second set of experiments, we again overexpressed the core PRC2, this time in conjunction with treatment of the HCT116 cells with Ac5S-GlcNAc. We then analyzed the UNC5A mRNA levels by RT-qPCR (Figure 5B). In agreement with the luciferase activity assay, overexpression of the core PRC2 led to a decrease in UNC5A mRNA and this was prevented by simultaneous treatment of cells with Ac5S-GlcNAc (Figure 5B, right panel). Taken together, these results demonstrate that *O*-GlcNAcylation regulates the PRC2-mediated repression of *UNC5A* in human colon cancer cells.

### 2.6. O-GlcNAcylation Drives the Recruitment of EZH2 onto the UNC5A Promoter

**Figure 6:**
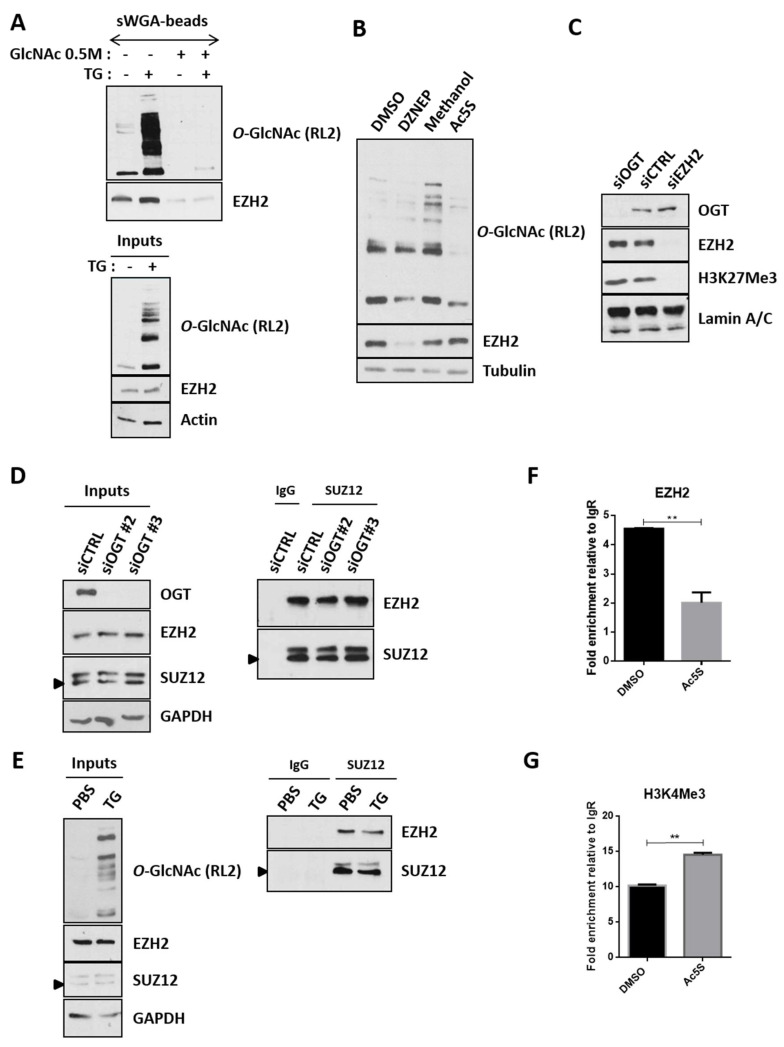
In colon cancer cells, *O*-GlcNAcylation neither influences EZH2 stability, methyltransferase activity nor its interaction with SUZ12 but drives the PRC2 recruitment onto the *UNC5A* promoter. (**A**) Enrichment of the *O*-GlcNAcylated proteins from Thiamet G-treated (1 µM final in PBS, 24 h) or non-treated cells were performed with sWGA-agarose beads as described in the experimental procedures section. In total, 25μg of whole cell lysates (Inputs) and bound proteins were analyzed by WB with anti-EZH2 and anti-*O*-GlcNAc (RL2) antibodies. Actin was used as a loading control. Data shown are representative of two independent experiments. (**B**) HCT116 cells were treated with the EZH2 destabilizing agent DZNEP (3-Deazaneplanocin A) (5 µM final in DMSO) or the OGT inhibitor Ac5S-GlcNAc (50 µM final in methanol) respectively for 48 h or 24 h. WB analyses were performed using the indicated antibodies. Tubulin was used as a loading control. Results are representative of at least three independent experiments. (**C**) HCTT116 cells were transfected either with non-target siRNA control (siCTRL) or siRNA targeting OGT (siOGT) or EZH2 (siEZH2, used as a positive control) for 72 h. Chromatin-bound proteins were extracted and WB analyses were performed using the indicated antibodies. Lamin A/C were used as a loading control. Results are representative of at least three independent experiments. (**D**–**E**) HCT116 cells were transfected with 2 different OGT-targeting siRNA (siOGT#2 or siOGT#3) or a non-relevant siRNA (siCTRL) for 72 h (**D**) or were treated with 1 µM Thiamet G for 24 h (**E**). The interaction between SUZ12 and EZH2 was then evaluated by co-immunoprecipitation experiments. Results are representative of three independent experiments. (**F**–**G**) HCT116 cells were treated with the OGT inhibitor Ac5S-GlcNAc (50 µM final in DMSO) for 24 h. Then binding of EZH2 (**F**) as well as presence of the H3K4Me3 activating mark (**G**) onto the *UNC5A* promoter were assessed by CUT&RUN experiments. Data shown represent the fold enrichment relative to the isotype control (Rabbit IgG) of a single experiment deposited in duplicate and is representative of two independent experiments. **p < 0.01: unpaired *t*-test.

We then investigated the mechanisms by which *O*-GlcNAcylation could influence the EZH2 mediated-repression of *UNC5A*. Indeed, regulation of EZH2 functions by its *O*-GlcNAcylation has been evidenced in several studies conducted in different cell lines but the exact roles of this glycosylation are not so clear. Indeed, while some studies argue for a role of *O*-GlcNAcylation in the regulation of EZH2 stability and catalytic activity [29,30,31], others propose that the glycosylation rather regulates EZH2 recruitment to some of its target genes such as *FOXC1* in breast cancer cells [32] or *IL-15* in muscle [33]. First, we checked the modification of endogenous EZH2 by *O*-GlcNAcylation in HCT116 cells by sWGA (succinyl Wheat Germ Agglutinin)-beads enrichment experiments (Figure 6A). Briefly, HCT116 cells were treated with Thiamet G for 24 h to maintain high *O*-GlcNAcylation levels in the cells (Figure 6A, bottom panel). Total cell lysates were incubated with sWGA-beads to extract *O*-GlcNAcylated proteins or, as a negative control, with sWGA-beads preincubated with free GlcNAc (Figure 6A, top panel). EZH2 was easily detected in the sWGA-enriched fractions (with or without TG) but not in the negative controls thus proving *O*-GlcNAcylation of EZH2 in HCT116 cells. Inhibition of OGT activity by Ac5S-GlcNAc did not affect EZH2 expression, whereas DZNEP, a well-known EZH2 destabilizing agent, did (Figure 6B). Similarly, the suppression of OGT expression by siRNA did not lead, as might be expected, to a decrease in EZH2 protein levels (Figure 4D and Figure 6C,D Inputs). In addition, increasing *O*-GlcNAcylation levels with Thiamet G did not lead to any variation in EZH2 expression (Figure 6A,E Inputs). All these results therefore do not argue for a stabilizing role of *O*-GlcNAcylation in our study model. *O*-GlcNAcylation also does not seem to influence the methyltransferase activity of EZH2 in this cell line since the inhibition of OGT activity or expression had no impact on the levels of H3K27Me3, the epigenetic mark deposited by the PRC2 complex (Figure 4E and Figure 6C). Among the EZH2 *O*-GlcNAcylation sites identified to date, Ser^313^ is located in the domain of interaction with SUZ12 [38]. Nevertheless, co-immunoprecipitation experiments between EZH2 and SUZ12 carried out in HCT116 cells under *O*-GlcNAcylation levels modulation conditions showed that this post-translational modification did not seem to influence the interaction between these two partners (Figure 6D,E). We finally analyzed whether *O*-GlcNAcylation could drive the recruitment of the PRC2 complex onto the *UNC5A* promoter. To test this hypothesis, we performed CUT&RUN experiments (an alternative method to ChIP that also enables mapping of protein-DNA interactions) under conditions of modulation of *O*-GlcNAcylation levels (Figure 6F,G). In this way, we showed that EZH2 was bound onto the *UNC5A* promoter in HCT116 cells and that inhibition of OGT with Ac5S-GlcNAc clearly diminished its binding (Figure 6F). On the contrary, OGT inhibition led to an increased binding of the H3K4Me3 activating mark on the *UNC5A* promoter (Figure 6G). So, taken together, our overall results demonstrate that in colon cancer cells, *O*-GlcNAcylation of EZH2 targets the PRC2 complex onto the *UNC5A* promoter to inhibit its transcription. 

## 3. Discussion

In recent decades, changes in Western lifestyle (increased sedentary not compensated by a decrease in caloric intake) have largely contributed to the increased incidence of CRC. In this regard, several epidemiological studies have shown that there is a higher risk factor for developing CRC in patients with metabolic syndrome, type-2 diabetes or obesity [16,17,18]. In addition to the well-known genetic origin of CRC, many studies have shown that colon carcinogenesis also involves alterations in the epigenetic regulation of genome. Furthermore, a growing number of studies tends to prove that the epigenome is able to integrate nutritional informations and it seems therefore obvious that nutritional intake, by modifying the epigenome, could influence the emergence and progression of CRC [1,2,3]. However, the underlying mechanisms are still insufficiently known. The nucleotide sugar UDP-GlcNAc, donor for the *O*-GlcNAc modification, is synthetized through the Hexosamine Biosynthesis Pathway (HBP) at the crossroad of glucose, amino acid, fatty acid and nucleotide metabolisms. UDP-GlcNAc and *O*-GlcNAcylation are therefore considered as sensors of the nutritional state of the organism [15] which can relay the effects of an excessive food supply, unbalanced diet, obesity and other metabolic problems. It has been previously shown that the expression of the core PRC2 complex (EZH2, EED and SUZ12) that catalyzes the deposit of the epigenetic repressive mark H3K27Me3 [26,27,28] as well as OGT and *O*-GlcNAcylation levels are increased during colorectal carcinogenesis [19,20,21,22]. Inversely, the expression of the tumor suppressor genes of the *UNC5* family is frequently downregulated in CRC in part through epigenetic mechanisms not fully deciphered [6,7,8,9,10]. In this study, in agreement with the fact that poor eating habits may influence colon carcinogenesis, we observed that consumption of a High Carbohydrate Diet (HCD) worsens colon carcinogenesis in mice. In this model, we found that the expression of UNC5A, UNC5B and UNC5C was diminished in tumors of mice only fed HCD but not of mice fed ND thus linking nutrition to their repression during the progression of CRC. We then tested the hypothesis that O-GlcNAcylation could be one of the molecular relays between this nutrition-dependent repression of UNC5A, UNC5B and UNC5C. Our in vivo *and* in vitro results argued against a direct correlation between *O*-GlcNAcylation levels and expression of UNC5B and UNC5C but strongly suggested a more direct link between *O*-GlcNAcylation and UNC5A expression. Mechanistically, we provide evidences that the *O*-GlcNAcylated form of EZH2 prevents the transcription of *UNC5A* in human colon cancer cells through aberrant *O*-GlcNAcylation and abnormal targeting of the PRC2 complex onto its promoter, thus linking nutrition to downregulation of *UNC5A* in CRC. However, further work will be required to understand the molecular link between nutrition and epigenetic regulation of the other members of the UNC5 family.

*O*-GlcNAcylation of EZH2 had already been described in several cell lines but the functions of this post-translational modification are still uncleared. In fact, some studies conclude that *O*-GlcNAcylation regulates EZH2 stability and methyltransferase activity [29,30,31,38] while others tempt to demonstrate that it rather steers the PRC2 complex onto specific loci [32,33]. Here, we confirmed the previously described *O*-GlcNAcylation of EZH2 in the colon cancer cell line HCT116. In our hands, *O*-GlcNAcylation does not seem to affect either stability or catalytic activity of EZH2 but appears to regulate the binding of EZH2 at specific loci including the *UNC5A* promoter in colon cancer cells.

UNC5 receptors belong to the family of the so-called “dependence receptors”. Such receptors initiate two opposite signaling pathways. When ligand (Netrin-1 for UNC5) is available, these receptors transduce a “positive signal” leading to cellular proliferation, differentiation, migration or survival. In absence of their ligand, they are still active but rather induce a “negative signal” that triggers caspase-dependent apoptosis [5]. Several studies have demonstrated that constitutive inhibition of the death signal induced by these receptors contributes to cell transformation. Conversely, their re-expression could represent a protective mechanism that limits tumor development through apoptosis induction of tumor cells, thus defining the members of the *UNC5* family as tumor suppressor genes. For example, in mice, inactivation of *UNC5C* is associated with increased intestinal tumor progression and decreased tumor cell apoptosis [8]. In human cancer cells, enforced expression of UNC5A, UNC5B and UNC5C inhibits cell-anchorage growth and invasion in a way related to their pro-apoptotic activity [7]. *UNC5A*, *UNC5B* and *UNC5D* are transcriptionally regulated by the tumor suppressor p53 and are involved in the p53-dependent apoptosis in response to DNA damages induced by conventional chemotherapeutic drugs like doxorubicin [39,40,41,42]. However, in an elegant paper, Paradisi et al. demonstrated, in a variety of cancer cell lines, that doxorubicin, 5-fluorouracil, paclitaxel and cisplatin treatments induced not only an increase of UNC5 receptors but also a concomitant p53-dependent increase of Netrin-1 thus preventing the pro-apoptotic action of UNC5 receptors [43]. In the same study, the authors also showed that interfering with Netrin-1 expression by siRNA or using two Netrin-1/Netrin-1 receptor inhibitors of interaction (TRAP-netrin^DCC^, TRAP-netrin^UNC5A^) potentiates doxorubicin induced cell death. According to these results, they proposed Netrin-1 upregulation as a survival mechanism of cancer cells in response to these drugs. Netrin-1 interference combined to conventional therapies has been then envisaged as a promising therapeutic approach for tumors resistant to chemotherapy. Interestingly, we observed that the siRNA knockdown of OGT led to increased *UNC5A* expression but had no effect on *Netrin-1* mRNA levels (data not shown). So, in the near future, it would be of great interest to go further into the mechanisms of transcriptional regulation of *UNC5A* by the *O*-GlcNAcylated form of EZH2 in response to chemotherapy and one could propose targeting with OGT/EZH2 interaction to enhance efficiency of chemotherapy in CRC.

## 4. Materials and Methods

### 4.1. AOM/DSS-Induced CRC Model

All the procedures were carried out according to the French guidelines for the care of experimental animals and the experimental procedure was approved by the Animal Care Committee of the French Research Ministry (Autor. APAFiS #1879-2018121918307521). Male C57BL/6JRj mice (n = 28; 8 weeks old; Charles River Saint-Germain sur l’Arbresle) were maintained under controlled room temperature, humidity and light (12/12 h light/dark cycle) with free access to food and tap water. Half of the mice were subjected to a Normal Diet (ND; standard laboratory chow; 16.1% protein, 3.1% lipid, 60.4% NFE (Nitrogen-Free Extract), 4.6% minerals and 3.9% fibers; A04; SAFE) and the other half to a High Carbohydrate Diet (HCD; 13.5% protein, 3.2% lipid, 76.8% NFE, 3.6% minerals, 0.7% cellulose and 1.1% starch; U8960P version 0002; SAFE) (Figure 1A). Thirty-nine days after the beginning of the two diets, we used the AOM/DSS method to induce inflammation-driven CRC [35]. Animals received a single intraperitoneal injection of azoxymethane (AOM, 10 mg/kg, A5486 Sigma) and they began to receive 2.5% dextran sulfate sodium (DSS, 0216011080 MP Biomedicals) in drinking water for 3 consecutive days followed by drinking water for 1 day. Mice were then submitted to three additional cycles of DSS (4 days with 2.5% DSS for the first one and 5 days with 1.5% DSS for the two others) with a resting period of 14 days between each cycle. Weight and blood glucose levels were monitored every 2 weeks during all the time course of the experiment. At the end of the experiment, tumor burden was monitored via endoscopy and colons were collected and washed in Phosphate Buffered Saline (PBS). Samples were kept at −80 °C for further RNA extraction. Part of the samples was also fixed in 10% buffered formalin overnight for further immunohistochemistry (IHC) analysis.

### 4.2. Assessment of Tumorigenesis Using Colonoscopy

Tumor developments were assessed using a high-resolution Karl Storz colonoscope (1.9 mm outer diameter; Tuttlingen, Germany) at the end of protocol (day 124). Mice were anesthetized using isoflurane for the duration of the procedure and closely monitored. For tumors assessment we followed the method described by Becker et al. [44]. Tumors observed from colonoscopy videos were counted, measured and categorized into size: grade 1 (very small but detectable tumor), grade 2 (tumor covering up to one eighth of the colonic circumference), grade 3 (tumor covering up to a quarter of the colonic circumference), grade 4 (tumor covering up to half of the colonic circumference) and grade 5 (tumor covering more than half of the colonic circumference).

### 4.3. Immunohistochemistry Staining and Quantification

After fixation, tissues were embedded in paraffin wax by automatic sample preparation system (LOGOS One, Milestone). Serial histological sections of 4 μm thickness were cut, deparaffinized and rehydrated. For antigen unmasking, sections were placed in 10 mM sodium citrate buffer pH 6.0 and incubated in a heat induced antigen retrieval chamber for 20 min at 121 °C. After washing, sections were blocked for 30 min with 5% BSA (Bovine Serum Albumin) in PBS. The following primary antibody was then incubated overnight at 4 °C: anti-*O*-GlcNAc (RL2) (NB300-524, Novus Biologicals) used at a concentration 1/1000. After washing, tissue sections were incubated 1 h at room temperature with the specified secondary antibody bound to HRP (Horseradish Peroxidase): Anti-Mouse IgG—Horseradish PeroxidaseLinked Species—Specific Whole Antibody (from sheep) (#NA931, GE Healthcare). Samples were washed with PBS and the signal was subsequently revealed with DAB (Diaminobenzidine) (#8059, Cell Signaling). Finally, tissue sections were counterstained with hematoxylin. Images were acquired with a DM5500B microscope (Leica Microsystems, Nanterre, France) and mucosal layers were photographed at a magnification of x20. The assessment of staining was performed using ImageJ software and the IHC score = staining intensity score (low:1, medium:2 and high:3) × % of surface staining. 

### 4.4. Cell Culture

HCT116 cells purchased from ATCC were maintained in Mc Coy’s 5A (modified) medium with Glutamax (Thermofischer Scientific) supplemented with 10% fetal calf serum and 1% ZellShield^TM^ (Biovalley). Cells were cultured at 37 °C in water-saturated 5% CO_2_ atmosphere.

### 4.5. Inhibitors

The EZH2 inhibitor, GSK343 (Sigma-Aldrich), was dissolved in dimethyl sulfoxide (DMSO) at 10 mM as stock solution and used at a final concentration of 5 µM. The EZH2 destabilizing agent, DZNEP (Sigma-Aldrich), was dissolved in DMSO at 10 mM as stock solution and used at a final concentration of 5 µM. The OGA inhibitor, Thiamet G (TG, Sigma-Aldrich), was dissolved in PBS at 1 mM as stock solution and used at a final concentration of 1 µM. The OGT inhibitor, Ac5S-GlcNAc (Ac5S, kind gift of GW. Hart and D. Vocadlo), was dissolved either in methanol or DMSO depending of their provenance at 50 mM as stock solution and used at a final concentration of 50 µM. Inhibitors were added to the cell culture medium for 24 h or 48 h.

### 4.6. Plasmids and Transfection

The expression vectors for Myc-EZH2, HA-EED and HA-SUZ12 have been previously described (24). Cells were seeded in 6 wells plates and transfected with 330 ng of each plasmid in 5 mL of complete culture medium by the Lipofectamine (Lipo2000, Invitrogen) method (4µL). Cells were harvested 48 h after transfection.

### 4.7. Small Interfering RNA

HCT116 cells were reverse-transfected with Lipofectamine RNAiMax (Invitrogen) according to manufacturer’s instructions using 5 nM small interfering RNA targeting OGT (siGENOME human OGT siRNA D-019111-01, Dharmacon), EZH2 (EZH2 siGENOME SMART Pool M-004218-03-0005, Dharmacon) or a scrambled control sequence (siCTRL; siGENOME RISC free control siRNA, Dharmacon) as previously described [19]. Seventy-two hours later, cells were harvested for RNA/protein extraction.

### 4.8. Quantitative RT-PCR

RNA was isolated using Nucleospin^®^ RNA mini spin kit (Macherey-Nagel) according to the manufacturer’s instructions. 1µg of total RNA was reverse transcribed using random primers and MultiScribeTM reverse transcriptase (Applied Biosystems). Real-time PCR analysis was performed by Power SYBR Green (Applied Biosystems) in a MX3005P fluorescence temperature cycler (Stratagene) according to the manufacturer’s instructions. Results were normalized with respect to *RPLP0* mRNA used as internal control. The primers used for the RT-qPCR analyses are summarized in Supplementary Table S1.

### 4.9. Total and Chromatin-Bound Proteins Extraction, Western Blotting and Antibodies

For total protein extraction, cells were lysed in RIPA (Radioimmunoprecipitation assay) buffer (10 mM Tris [pH 7.4], 150 mM NaCl, 1 mM EDTA, 1% Triton X-100, 0.5% sodium deoxycholate, 0.1% SDS and proteases inhibitors added at the time of preparation). For chromatin-bound proteins extraction, subcellular fractionation was performed as described in Füzesi-Levi et al. [45]. Protein concentration was determined using the Micro BCA (Bicinchoninic acid) Protein Assay Kit (Thermofisher Scientific). Equal amounts of proteins were separated by SDS-PAGE and transferred onto nitrocellulose membranes (GE Healthcare). After 1 h of blocking in PBSM (PBS with 5% milk), the membranes were incubated overnight at 4 °C with specific primary antibodies in PBSTM (PBSM with 0.1% Tween) and washed three times with PBSN (PBS with 0.1% NP-40). The membranes were next incubated for 1 h at room temperature with secondary antibodies coupled to peroxydase (Amersham) in PBSM, washed three times in PBSN and revealed by chemiluminescence.

Mouse monoclonal anti-*O*-GlcNAc (RL2) was purchased from Life technologies (MA1072). Rabbit polyclonal anti-OGT (DM17) was purchased from Sigma-Aldrich (#O6264). Mouse monoclonal anti-tubulin (sc-23948), mouse monoclonal anti-GAPDH (sc-32223), rabbit polyclonal anti-lamin A/C (sc-20681), rabbit polyclonal anti-actin (sc-1616-R) and rabbit polyclonal anti-Myc (sc-789) were purchased from Santa Cruz Biotechnology. Rabbit polyclonal anti-EZH2 (#5246) and rabbit polyclonal anti-H3K27Me3 (#9733) were purchased from Cell signaling. Rabbit polyclonal anti-SUZ12 was purchased from Abcam (ab12073). Mouse monoclonal anti-HA was purchased from Biolegend (MMS-101P). Anti-mouse IgG-HRP was purchased from GE Healthcare (NA931V). Donkey anti-rabbit IgG-HRP was purchased from Millipore (AP182P).

### 4.10. Luciferase Promoter Activity Assays

The pGl3-UNC5A promoter construct has been kindly provided by A. Paradisi (P. Mehlen team, Cancer research center of Lyon, Lyon, France). HCT116 cells, seeded in 12-wells plates, were transfected with 200 ng of the *UNC5A* promoter activity reporter plasmid and 25 ng of β-galactosidase reporter using Lipofectamine 2000 (Invitrogen) according to the manufacturer’s instructions. Thus, 48 h after transfection, cells were lysed in Luciferase assay buffer (25 mM glycyl glycine [pH 7.8], 15 mM MgSO_4_, 4 mM EGTA, 1% Triton X-100). Luciferase and β-galactosidase activities were measured by using, respectively, beetle luciferine (Promega) and the Galacto-light kit (Tropix) with a Berthold chemiluminometer. After normalization to the β-galactosidase activity, the data were expressed as the fold-change *UNC5A*-luciferase activity relative to control, which was given an arbitrary value of 1. A fraction of cell lysates was also subjecting to Western Blot (WB) analyses to ensure efficiency of cells treatments with siRNA or inhibitors.

### 4.11. Enrichment of O-GlcNAc-Bearing Proteins with sWGA Immobilized on Agarose BEADS

Cells were lysed in RIPA buffer and protein concentration was determined. For each condition, 2 mg of proteins were incubated either with 50 µL of succinylated-Wheat Germ Agglutinin (sWGA) agarose beads (Vector Laboratories) for 2 h at 4 °C or with sWGA beads beforehand incubated with 0.5 M free GlcNAc (to control the specificity of the reaction). sWGA-bound proteins were collected and washed three times with washing buffer (10 mM Tris [pH 7.4], 150 mM NaCl, 0.5 mM EDTA, 1% Triton X-100, 0.5% sodium deoxycholate, 0.2% SDS). Proteins were then eluted from the beads in 2X Laemmli buffer (50 mM Tris-HCl pH6.5, 2.5% SDS, 5% 2-mercaptoethanol, 10% glycerol and a hint of bromophenol blue) and resolved by SDS-PAGE.

### 4.12. Co-Immunoprecipitation

Twenty-four hours or 72 h respectively after Thiamet G treatment and siRNA transfection, cells were rinsed with cold PBS and lysed with 1 mL of cold IPH (Immunoprecipitation homogeneization) buffer (50 mM Tris pH8, 150 mM NaCl, 5 mM EDTA and 0.5% NP40) supplemented with proteases inhibitors. Cells were put on ice for 15 min and scraped before being placed in rotation at 4 °C for 15 min. Cell lysates were briefly sonicated (22%—2 s). After a centrifugation at 14,000 rpm for 15 min at 4 °C, the supernatant was collected and the protein amount was determined with the Micro BCA Protein Assay Kit (Thermofisher Scientific). For each condition, 2 mg of proteins were then pre-cleared with 15 µL of protein A/G sepharose beads (GE Healthcare) and placed in rotation at 4 °C at least 1 h. Following this pre-clearing stage, cells lysates were incubated with 4µg of SUZ12 antibody (ab12073, Abcam) or with 4 µg of Normal Rabbit IgG (#2729, Cell Signaling) on a rotator overnight at 4 °C. Thereafter, 20 µL of protein A/G sepharose beads were added and placed in rotation 1 h at 4 °C. After three washes with IPH buffer, bound proteins were eluted by boiling in 2X Laemmli buffer and resolved by SDS-PAGE.

### 4.13. Cut&Run Experiments

Protein-DNA interactions were analyzed using the CUT&RUN (Cleavage Under Targets & Release Using Nuclease) Assay Kit (#86652, Cell Signaling Technology) following the supplier’s instructions. For each reaction, 250,000 cells were used. Positive (H3K4Me3) and negative (Rabbit mAb IgG) controls were performed using the antibodies provided in the kit. The EZH2 (#5246, Cell Signaling Technology) antibody was used at a dilution of 1/100. For DNA purification, the High Pure PCR Template Preparation Kit (#11796828001, Roche) was used according to the manufacturer’s procedures except for the elution which was done in 50 µL buffer. Finally, binding of EZH2 and presence of the activating mark H3K4Me3 onto the *UNC5A* promoter was quantified by qPCR using the following primers: *UNC5A forward*: CCCTGACACCGTGTACATTCA, *UNC5A reverse*: TCACCACCTTCTGGTTTGGG. 

## 5. Conclusions

In conclusion, we provided evidences that the nutritional sensor *O*-GlcNAcylation represents one of the molecular relays between nutritional disorders and the epigenetic downregulation of key tumor suppressor genes driving the progression of colorectal carcinogenesis including *UNC5A*. In the near future, it will therefore be interesting to investigate whether strategies targeting *O*-GlcNAcylation could represent a new therapeutic hope in the treatment of colorectal cancer. 

## Supplementary Materials

The following are available online at https://www.mdpi.com/2072-6694/12/11/3168/s1, Figure S1: Blood glucose levels; Figure S2: LS174T cells were maintained in EMEM (Lonza) supplemented with 10% fetal calf serum and 1% ZellShieldTM (Biovalley). Cells were cultured at 37 °C in water-saturated 5% CO_2_ atmosphere. LS174T cells were transfected either with non-target siRNA control (siCTRL), OGT siRNA (siOGT), EZH2 siRNA (siEZH2) or with a combination of both for 72 h. Cells were harvested either for total proteins or RNA extraction; Table S1: RT-qPCR primers used in this study.
