# Supplementary material for: O-GlcNAcylation Links Nutrition to the Epigenetic Downregulation of UNC5A during Colon Carcinogenesis
Source: Cancers (Basel). 2020 Oct 28;12(11):3168. doi: 10.3390/cancers12113168 (PMC7693889; doi:10.3390/cancers12113168)
Supplement: Supplementary file 1 [file cancers-12-03168-s001.zip › supplementary data decourcelle et al cancers 2020 submitted version.pptx]

## Slide 1
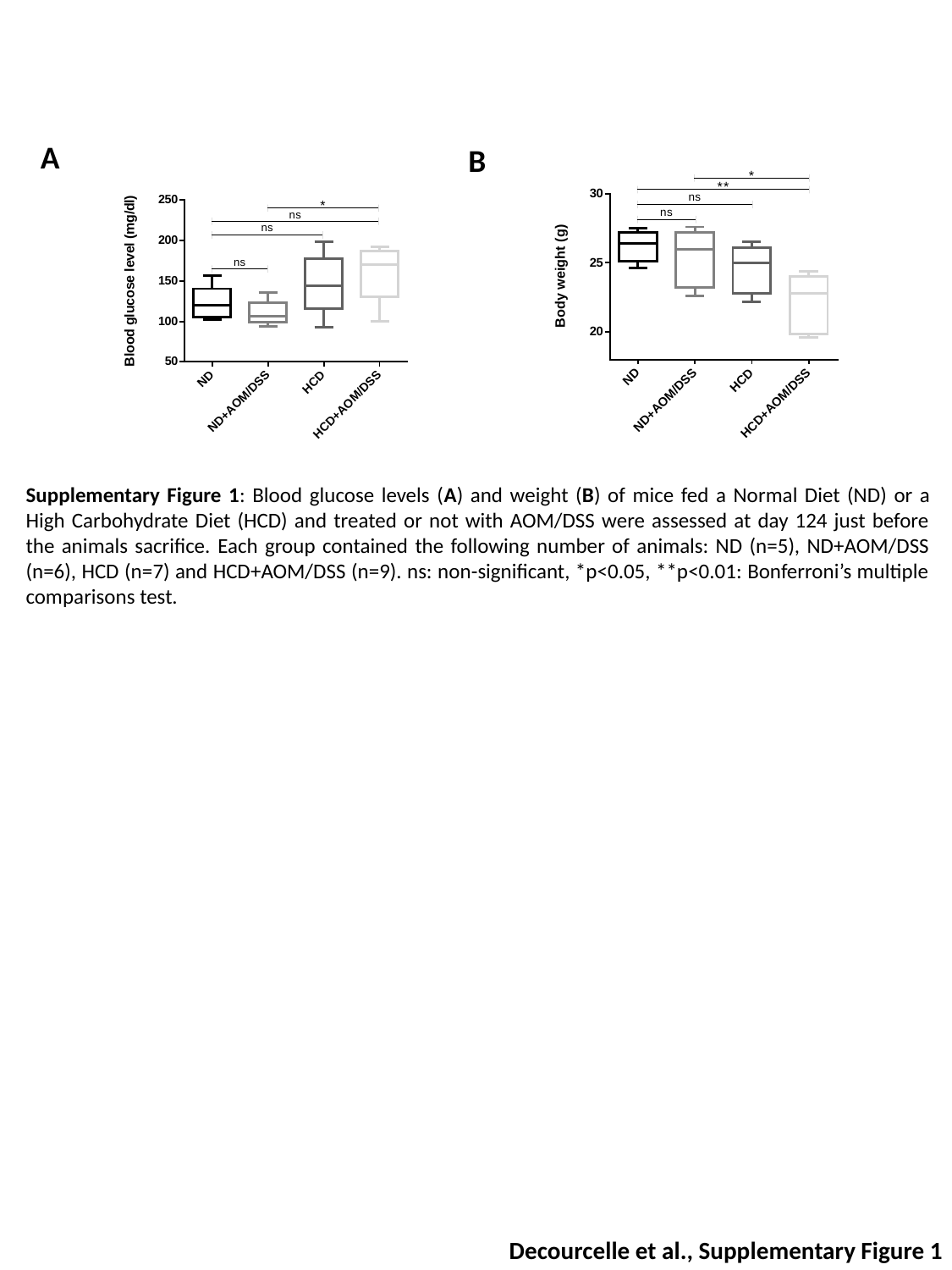

A
B
Supplementary Figure 1: Blood glucose levels (A) and weight (B) of mice fed a Normal Diet (ND) or a High Carbohydrate Diet (HCD) and treated or not with AOM/DSS were assessed at day 124 just before the animals sacrifice. Each group contained the following number of animals: ND (n=5), ND+AOM/DSS (n=6), HCD (n=7) and HCD+AOM/DSS (n=9). ns: non-significant, *p<0.05, **p<0.01: Bonferroni’s multiple comparisons test.
Decourcelle et al., Supplementary Figure 1

## Slide 2
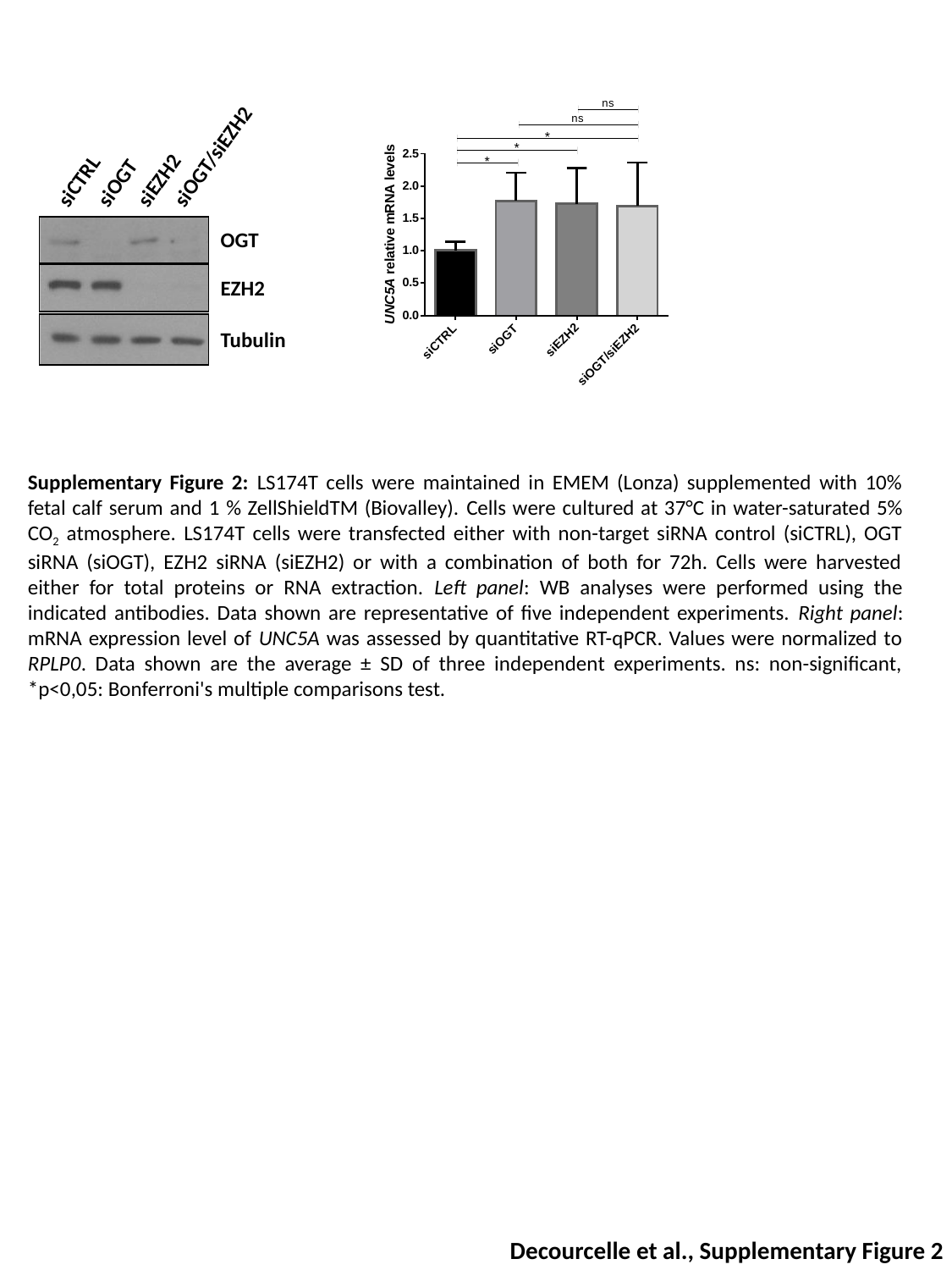

siOGT/siEZH2
siCTRL
siOGT
siEZH2
OGT
EZH2
Tubulin
Supplementary Figure 2: LS174T cells were maintained in EMEM (Lonza) supplemented with 10% fetal calf serum and 1 % ZellShieldTM (Biovalley). Cells were cultured at 37°C in water-saturated 5% CO2 atmosphere. LS174T cells were transfected either with non-target siRNA control (siCTRL), OGT siRNA (siOGT), EZH2 siRNA (siEZH2) or with a combination of both for 72h. Cells were harvested either for total proteins or RNA extraction. Left panel: WB analyses were performed using the indicated antibodies. Data shown are representative of five independent experiments. Right panel: mRNA expression level of UNC5A was assessed by quantitative RT-qPCR. Values were normalized to RPLP0. Data shown are the average ± SD of three independent experiments. ns: non-significant, *p<0,05: Bonferroni's multiple comparisons test.
Decourcelle et al., Supplementary Figure 2

## Slide 3
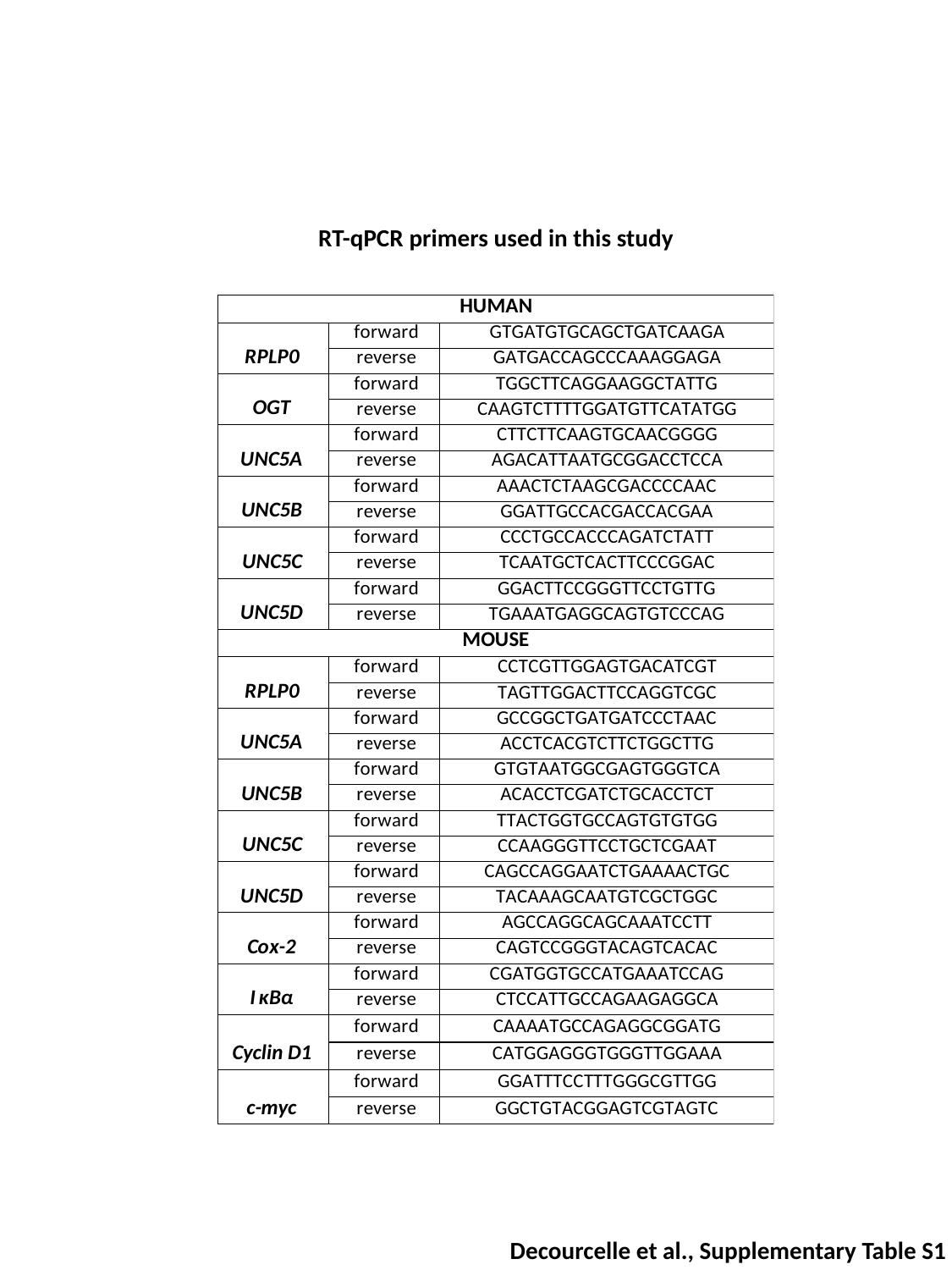

RT-qPCR primers used in this study
Decourcelle et al., Supplementary Table S1
